# Supplementary material for: Long-Term Outcome and Rejection After Allogeneic Uterus Transplantation in Cynomolgus Macaques
Source: J Clin Med. 2019 Oct 1;8(10):1572. doi: 10.3390/jcm8101572 (PMC6833021; doi:10.3390/jcm8101572)
Supplement: Supplementary file 1 [file jcm-08-01572-s001.pdf]

Supplemental Table S1. MHC genes of four recipient and donor pairs.

Case 1			Case 2		
	**Donor**	**Recipient**		**Donor**	**Recipient**
ABO Type	AB	AB	ABO Type	B	B
Mafa-A	A1 *003:03	A1*040:01	Mafa-A	A1*018:06	A1*057:01
	A4*14:03/04/13	A1*056:03		A1*043:10	A1*069:01
	A4*14:17/20	A2*05:22/55		A1*086:03	A4*14:03/04/13
		A4*14:03/04/13		A2*05:34	A6*01:05_New
		A6*01:04		A4*14:01/08/10	
				A8*01:01	
Mafa-B	B*003:02_New	B*007:01/07/09/11	Mafa-B	B *007:01/07/09/11	B*013:16
	B*007:01/07/09/11	B*021:01/05/07		B*013:11	B*061:01
	B*011:02	B*028:03/05/06		B*039:01/03	B*061:05
	B*039:01/03	B*039:01/03		B*046:13	B*068:04/10
	B*051:05/08	B*051:05/08		B*051:05/08	B*074:01N/05N
	B*060:04/13/19	B*060:04/13/19		B*060:04/13/19	B*074:02
	B*072:05/09_New	B*068:02		B*060:14	B*124:01/03
	B*079:02	B*072:05/09		B*088:02/04	B*137:06/07/08
	B*088:02	B*079:06		B*114:01/04	
		B*088:02			
		B*124:01/02/04			
		B*144:04_New			
Mafa-DRB	DRB*W001:01	DRB*W001:01	Mafa-DRB	DRB*W001:01	DRB1*10:09
	DRB*W003:02	DRB*W003:08		DRB*W003:02	DRB3*04:04
	DRB1*03:03/30	DRB*W007:04:02		DRB*W027:06	DRB3*04:11
	DRB1*03:08	DRB1*03:07		DRB1*03:03/30	
	DRB1*10:04	DRB1*04:07		DRB3*04:06	
		DRB1*10:11			
Mafa-DQA1	DQA1*01:03	DQA1*01:12	Mafa-DQA1	DQA1*01:03	DQA1*05:13
	DQA1*01:08	DQA1*05:03			DQB1*24:01
Mafa-DQB1	DQB1*06:01	DQB1*06:13/44/45	Mafa-DQB1	DQB1*06:07	DQB1*15:03
	DQB1*06:16	DQB1*15:03		DQB1*06:16	DQB1*24:01
Mafa-DPA1	DPA1*02:11/22	DPA1*02:30	Mafa-DPA1	DPA1*02:09	DPA1*02:07
	DPA1*09:01:01	DPA1*02:36		DPA1*04:01	
Mafa-DPB1	DPB1*15:02/08	DPB1*01:02/11/14	Mafa-DPB1	DPB1*03:03/04/05	DPB1*18:02
	DPB1*17:01	DPB1*09:01		DPB1*15:01/13	
**Case 3**			**Case 4**		
	Donor	Recipient		Donor	Recipient
ABO Type	B	B	ABO Type	B	B
Mafa-A	A1*041:01	A1*027:01	Mafa-A	A1*022:04	A1*007:03
	A1*091:02/03	A1*045:04		A1*063:01/02/03	A1*089:03/05
	A4*14:01/08/10			A4*14:03/04/13	A3*13:04/08
	A5*30:03/06			A*14:01/08/10_New	A8*01:01
Mafa-B	B*007:05/08/10	B*021:01/05/07	Mafa-B	B*007:01/07/09/11	B*048:06_New
	B*030:01/03/07/16/17/18	B*028:04		B*017:02	B*060:03/20/21/22/24/28

	B*046:04/09/17	B*030:01/03/07/16/17/18		B*018:01	B*063:02
	B*060:04/13/19	B*030:08N		B*030:01/03/07/16/17/18	B*068:05/08/12
	B*068:03	B*051:13		B*044:01	B*099:01
	B*068:06/07/11/13	B*060:03/20/21/22/24/28		B*051:12	B*108:01
	B*082:01/06	B*068:02		B*061:03	
	B*098:16/17	B*070:01/02		B*072:05/09	
	B*144:01	B*082:05		B*085:01	
	B*145:01	B*089:01			
	B*149:01				
Mafa-DRB	DRB*W001:01	DRB*W002:08	Mafa-DRB	DRB*W001:01	DRB*W001:07
	DRB*W037:02	DRB*W006:01/06/07		DRB*W003:02	DRB*W006:02
	DRB*W064:01	DRB*W037:02		DRB*W004:04/10/11	DRB*W006:03
	DRB1*03:03/30	DRB1*03:23		DRB1*03:03/30	DRB*W049:01
	DRB1*04:13/16	DRB1*04:13/16			DRB1*10:02
				DRB3*04:03:01	DRB3*04:02
Mafa-DQA1	DQA1*05:04	DQA1*01:03	Mafa-DQA1	DQA1*01:03	DQA1*24:10
		DQA1*05:04		DQA1*23:01	DQA1*05:03
Mafa-DQB1	DQB1*17:02	DQB1*06:19	Mafa-DQB1	DQB1*06:16	DQB1*16:01
	DQB1*17:05/10	DQB1*17:05/10		DQB1*18:04	DQB1*18:07/26
Mafa-DPA1	DPA1*02:09	DPA1*02:01/27	Mafa-DPA1	DPA1*06:01/05	DPA1*02:01/27
	DPA1*06:01/05	DPA1*04:01		DPA1*10:03	DPA1*04:02
Mafa-DPB1	DPB1*01:02/11/14	DPB1*01:01/07/13	Mafa-DPB1	DPB1*01:02/11/14	DPB1*01:01/07/13
	DPB1*15:01/13	DPB1*02:04/06		DPB1*18:01/03	DPB1*03:03/04/05

Supplemental Table S2. Antibodies for flow cytometry.

Antibody	Dye	Clone	Vendor	Cat#	Isotype control	Clone	Vendor	Cat#
**CD3**	PE-Cy7	SP34-2	BD	557749	Mouse IgG1 κ	MOPC-21	BD	557872
**CD4**	FITC	OKT4	BioLegend	317408	Mouse IgG2b κ	MPC-11	BioLegend	400310
**CD4**	APC	OKT4	BioLegend	317416	Mouse IgG2b κ	MG2b-57	BioLegend	401210
**CD8**	BV510	RPA-T8	BioLegend	301048	Mouse IgG1 κ	MOPC-21	BioLegend	400172
**CD16**	V450	3G8	BD	561246	Mouse IgG1 κ	X40	BD	561504
**CD20**	PE	2H7	BioLegend	302306	Mouse IgG2b κ	MPC-11	BioLegend	400314
**FOXP3**	PE	236A/E7	eBioscience	72-5774-40	Mouse IgG1 κ	MOPC-21	BD	555749

Abbreviations: Cat#: Catalog number, PE-Cy7: phycoerythrin-cyanin 7, FITC: fluorescein isothiocyanate, APC: Allophycocyanin, BV510: Brilliant Viole 510, V450: Violet 450, PE: phycoerythrin, BV421: Brilliant Violet 421, BD: Becton, Dickinson and Company including BD bioscience and BD pharmingen.
